# RESIDENTS’ PERSPECTIVES ON PROGRAM IMPACT OF A GROUP-BASED MIND–BODY INTERVENTION IN CCRCS

**DOI:** 10.1093/geroni/igae098.3412

**Published:** 2024-12-31

**Authors:** Calli Mitchell, Riley Psenka, Reid Anctil, Madeleine Scully, Barrett Laird, Elyse Park, Lara Traeger

**Affiliations:** Massachusetts General Hospital, Boston, Massachusetts, United States; Massachusetts General Hospital, Boston, Massachusetts, United States; Massachusetts General Hospital, Boston, Massachusetts, United States; Massachusetts General Hospital, Boston, Massachusetts, United States; Massachusetts General Hospital, Boston, Massachusetts, United States; Massachusetts General Hospital, Boston, Massachusetts, United States; Massachusetts General Hospital, Boston, Massachusetts, United States

## Abstract

Older adults in continuing care retirement communities (CCRCs) may experience critical stressors related to biological and social aging. Few interventions have been established to assist residents with managing life stressors, daily functioning, and sense of community. We conducted an analysis of exit feedback from intervention participants in a randomized trial of group-based stress management skills training (SMART-Relaxation Response Resiliency Program [SMART-3RP]; 9 weekly sessions delivered by a community clinician) versus waitlist control for independent living residents at nine CCRCs across the United States (N=288; 8/2022-1/2023). SMART-3RP is an evidence-based mind-body resiliency program that integrates relaxation response training with cognitive-behavioral and positive psychology principles. Intervention participants (n=148) completed a post-intervention questionnaire including 3 categorical items and open-text fields to evaluate program helpfulness with managing life stressors, daily functioning, and sense of community. Responses were evaluated using descriptive statistics and qualitative content analysis. 131/148 (88.5%) intervention participants provided feedback (77% female, M age=81 years [range=66-93 years], 91.5% White non-Hispanic). Most endorsed that the program helped manage life stressors (82.4%) and daily functioning (77.9%); participants further identified benefits of learning or strengthening skills for practicing gratitude, relaxation, emotion regulation, mindful awareness, self-compassion, and cognitive restructuring. Over half (56.9%) felt the program enhanced sense of community; residents reflected the value of connecting with group leaders and/or other group members and increasing motivation to engage in other community social and physical activities. Findings support that community residents valued participation in a group-based intervention to enhance skills for managing life stressors, daily functioning, and social connection.

